# Therapeutic Study of Thermosensitive Hydrogel Loaded with Super-Activated Platelet Lysate Combined with Core Decompression Technology for the Treatment of Femoral Head Necrosis

**DOI:** 10.1155/2021/7951616

**Published:** 2021-06-25

**Authors:** Zhipeng Huang, Zhe Zhao, Jun Lang, Wantao Wang, Yinsheng Fu, Wenbo Wang

**Affiliations:** ^1^The First Affiliated Hospital of Harbin Medical University, 23 You Zheng Street, Harbin 150001, China; ^2^Southern University of Science and Technology Hospital, 6019 Liuxian Avenue, Xili, Nanshan District, Shenzhen 518000, China; ^3^Tianqing Stem Cell Co., Ltd., Jubao Second Road, Science and Technology Innovation City, Songbei District, Harbin 150000, China

## Abstract

Super activated platelet lysate (sPL) is a derivative of platelet-rich plasma (PRP) that contains high levels of several growth factors. In this study, we synthesized a temperature-sensitive hydrogel that contained temperature-sensitive Poly(DL-lactide-glycolide-glycolide acid) (PLGA), SrCl_2_, and HA, and loaded it with different concentrations of sPL. The hydrogel showed satisfactory encapsulation efficiency and release of the growth factors in a sustained manner, indicating its suitability as a drug carrier. The sPL-loaded hydrogel was inserted into the necrotic femoral head of a rat model and core decompression was applied and resulted in significantly accelerated bone repair and regeneration. Therefore, encapsulation of sPL in a hydrogel scaffolding may be an effective strategy for treating femoral head necrosis.

## 1. Introduction

Femoral head necrosis (ONFH) is characterized by osteocytic necrosis and bone marrow necrosis as a result of insufficient or a complete lack of blood supply to the subchondral bone. There are currently 30 million diagnosed cases of ONFH worldwide, of which 8.12 million are in China alone, and the rate of incidence has been increasing annually [1]. A recent multicenter study conducted on ONFH patients in China found that steroid use was identified as the causative factor in 26.35% of males patients and 55.75% of female patients [2]. Long-term use of high-dose glucocorticoids (GC) may affect the differentiation of cells in the femoral head and alter bone metabolism, thereby decreasing the angiogenic activity of the femoral head and triggering ischemic hypoxia.

In the absence of an effective method of treatment, ONFH can progress into subchondral bone collapse in 80% of patients within 1-3 years. Subchondral bone collapse causes resulting in considerable pain and impaired hip joint function and will eventually require total hip arthroplasty (THA). However, since THA is not the best option for younger patients, action needs to be taken as early as possible to slow down the progression of ONFH and delay the age of joint replacement.

Core decompression (CD) is a joint-preserving surgery [3] that is suitable for the early stage ONFH patients with an intact joint surface [4]. It can reduce intramedullary pressure on the femoral head, accelerate bone regeneration that may form a cavity after core decompression, and reverse femoral head necrosis [5], thereby delaying the progression of the disease and preventing femoral head collapse. However, 37% of patients that undergo core decompression therapy will progress to femoral head collapse [4].

Platelet-rich plasma (PRP) can accelerate bone formation by restoring osteoblast proliferation and activating pathways that promote angiogenesis and osteogenesis [6], which is a strategy that can be used to the pathological progression of ONFH. Growth factors in PRP can promote cartilage formation [7] and osteogenesis [8] and can therefore alleviate ONFH [9]. In addition, platelet lysate (PL) growth factors also promote the chemotactic migration of various cells [10, 11]. PL can be incorporated into biological scaffolds that can retain its beneficial effects for a longer period [7, 12] based on the preparation method, activation, initial platelet concentration, and donor [12, 13]. Super-active platelet lysate (sPL) is prepared from PRP via ultralow temperature freeze-thawing. It is enriched in bioactive factors that can promote tissue regeneration and vascular remodeling. However, the short half-life of the growth factors in sPL limits their biological effects *in vivo*. In addition, high dosageses and/or the frequent administration of sPL is not economically viable, and may lead to toxic side effects. The controlled release of sPL loaded with hydrophilic macromolecules such as growth factors can significantly improve its efficacy *in vivo*.

Polymers with a high molecular weight [14], such as poly lactic-co-glycolic acid (PLGA), can be used as effective *in vivo* drug delivery systems due to their biocompatibility and biodegradability. The polymer material can protect sPL from the tissue microenvironment, leading to controlled release. Bone is a natural organic-inorganic-inorganic composite material that is mainly composed of collagen and hydroxyapatite (HA, Ca_10_(PO_4_)_6_(OH)_2_)). A variety of organic-inorganic composite materials that can mimic the composition and structure of bones have been developed. Strontium (Sr) is a trace element found in the human body that promotes bone formation and the healing of osteoporotic tissues. Therefore, we designed a composite hydrogel that consisted temperature-sensitive PLGA, the bone mineral hydroxyapatite (HA), and strontium [15, 16]. However, the hydrogel can flow into other parts of the defect, and the properties of the hydrogel need to be changed to prevent the hydrogel from flowing out.

Temperature-sensitive PLGA/HA/SrCl_2_ hydrogel loaded with sPL was used to reconstruct the degenerated bone tissue in combination with CD surgery. sPL was slowly released from the hydrogel in a temperature-sensitive manner and resulted in accelerated bone repair after core decompression of the femoral head and ONFH. Therefore, our study has laid the foundation for the clinical application of heat-sensitive hydrogel materials, and also for a considerable reduction in the cost of treating ONFH patients.

## 2. Materials and Methods

### 2.1. Materials

Temperature-sensitive PLGA was purchased from Jinan Daigang Biomaterial Co., Ltd, SrCl2 was purchased from Sinopharm Chemical Reagent Co. Ltd., HA was purchased from Aladdin, lipopolysaccharide was purchased from Sigma-Aldrich Inc. (USA), while Methylsulfonate was purchased from Pfizer Pharmaceuticals (Hangzhou, China).

### 2.2. sPL Preparation

sPL was isolated from human blood via ultralow temperature freezing, as previously described [17]. In brief, PRP was extracted by centrifuging fresh whole blood. The PRP was ultralow-frozen using melt preparation and patented cytokine culture technologies, sPL can be efficiently induced, activated, and cultivated.

### 2.3. Synthesis and Characterization of Hydrogels

The temperature-sensitive PLGA\SrCl_2_ and HA were mixed at a ratio of 94 : 5 : 1, and the mixture was added to 0, 250, and 500 *μ*l of sPL placed in a magnetic stirrer. The resulting PLS0, PLS1, and PLS2 hydrogels were lyophilized and sprayed with gold, and their morphology was observed under a scanning electron microscope (SEM) (Japan Electronics Co. Ltd.).

To measure the sustained release of bioactive factors from the PLS hydrogels created, the latter were placed in clean vials (*n* = 4 per group) and completely submerged in 4 ml of simulated body fluid (SBF). The vials were then sealed and incubated in a water bath, and the aliquots in the medium were collected on days 3, 6, 9, 12, 15, 18, 21, 24, 27, and 30. The concentration of VEGF and TGF-*β* was measured using specific ELISA kits (Jingmei, Jiangsu).

Preweighed lyophilized gels were incubated in deionized water at 30°C, 34°C, 38°C, and 42°C for 1 hour to measure hydrogel swelling. The swollen gels were retrieved, blotted to remove excess water, and weighed. The colloidal water content (SR) was calculated using the formula: (*W*1 − *W*0)/*W*0, where *W*0 and *W*1 indicate dry weight and temperature weight, respectively.

### 2.4. *In Vivo* Experiments

#### 2.4.1. Establishment of an ONFH Model and Treatment

All animal experiments were conducted in accordance with the guidelines on the humane use and care of animals formulated by the National Institutes of Health, all experimental animals are taken care of, and all operations on animals are approved by the Experimental Animal Use and Welfare Ethics Committee of the First Affiliated Hospital of Harbin Medical University (Ethical approval number:2019029).

Male SD rats weighing 280-300 g were reared at the animal center of the First Affiliated Hospital of Harbin Medical University and fed *ad libitum* on a standard laboratory diet and water. A total of 27 rats were intravenously injected with 10 *μ*g/kg lipopolysaccharide (LPS), followed by 24 h later with three intramuscular injections of 20 mg/kg methylsulfonate (MPS) then at 24 h intervals. Osteonecrotic lesions first appeared two weeks after the procedure and continued to appear until 6 weeks after the procedure [18]. Three of the rats were euthanized, and tissue samples were collected for CT and histopathological examination. Once a diagnosis of ONFH was confirmed, the other rats were randomly assigned to the PLS0, PLS1, and PLS2 groups (*n* = 8). The rats were anesthetized using 3% pentobarbital sodium, and the right hip was exposed using an anterior and posterior approach while preserving the main blood vessels in the femoral head. The hip joint was prevented from shifting by cutting the switch capsule, and the femoral head and neck were exposed. Decompression was performed from the greater trochanter to the core of the femoral head using a drill, and the lesion was filled with PLS. The implanted region was covered with bone wax, and the wound was closed. All animals were intramuscularly injected with gentamicin (4 mg/kg) before and after the operation to prevent any wound infections.

#### 2.4.2. Radiological Analysis

Four animals from each group were sacrificed to be used for the Micro-CT analysis on the 4^th^ and 12^th^ weeks postsurgery. Changes in the femoral head were monitored using a QuantumGX CT imaging system (PerkinElmer, USA). An isotropic 20 mm voxel pitch dataset was obtained using a total rotation of 360° and step length of 0.5° under a power of 80 kV, and computer-generated three-dimensional model of the femoral head was constructed.

#### 2.4.3. Histopathological Analysis

Animals from each group were used for the histological observations made on the 4^th^ and 12^th^ weeks after surgery. The tissue samples were fixed using 4% paraformaldehyde for a period of 1 week and decalcified using 20% EDTA solution for a period of a month. Then, the samples were dehydrated using an alcohol gradient and clarified using xylene. Thereafter, the tissues were embedded in paraffin and cut into 4 *μ*m thick sections, followed by hematoxylin and eosin (HE), Masson, and ALP staining, as per standard protocols.

#### 2.4.4. Tunel Assay

The tissue sections were incubated with the Tunel reagent using the specific Tunel kit as per the manufacturer's instructions. The color was developed by applying 0.03% DAB for 5 minutes, and the slides were counterstained with hematoxylin. The number of Tunel-positive cells were counted in four random microscopic fields at 200x magnification. The apoptosis rate (%) was calculated as the ratio of the number of Tunel-positive cells to the total number of cells.

#### 2.4.5. Immunohistochemistry

The tissue sections were probed using primary antibodies against type I collagen and CD31, followed by a secondary antibody with/out Cy5 conjugate. After counterstaining with hematoxylin, the slides were observed under a fluorescence microscope (Leica, Mannheim, Germany) or a light microscope (Leica, Mannheim, Germany).

### 2.5. Statistical Analysis

Data are expressed as mean ± standard deviation (SD). One-way analysis of variance (ANOVA) was used to compare data between the groups. A *P* value of <0.05 was considered to be statistically significant.

## 3. Results and Discussion

### 3.1. Characterization of the Hydrogels

**Figure 1:**
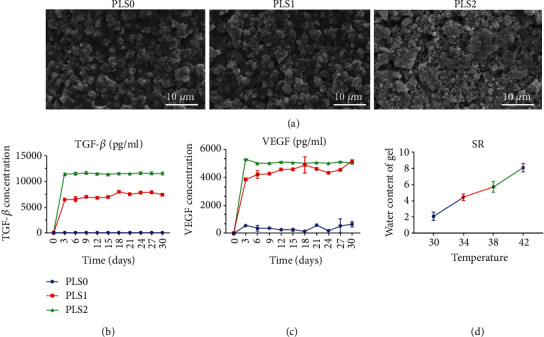
(a) Representative SEM images showing the structure of lyophilized thermosensitive hydrogel. (b, c) Release of growth factors from the loaded sPL hydrogel. (d) Temperature-dependent change in water content.

We loaded sPL into a temperature-sensitive hydrogel for sustainable release and stronger therapeutic effects. As shown in Figure 1(a), the freeze-dried hydrogel had a partially porous structure, and the incorporation of sPL decreased the size of the particles.

The polymer hydrogel steadily released TGF-*β* and VEGF over a period of 30 days (Figures 1(b) and 1(c)) depending on the amount of encapsulated sPL. As expected, there was no obvious release of growth factors from PLS0. In addition, the hydrogel swelled to 2.1, 2.8, and 3.9 times to its volume at 30°C when heated to 34°C, 38°C, and 42°C, respectively (Figure 1(d)).

### 3.2. The sPL-Loaded Hydrogel Alleviated Osteonecrosis and Promoted Bone Formation

**Figure 2:**
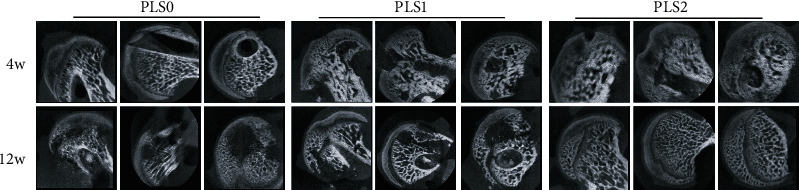
Representative coronal, transverse and sagittal micro-CT scans of the different treatment groups.

An ONFH model was established using LPS and MPS, as previously described by Wu et al. [19]. Imaging and histological tests clearly demonstrated a diagnosis of osteonecrosis at 6 weeks, along with bone marrow necrosis, abnormal fat distribution, and increased internal pressure of the femoral head. The core decompression channel for femoral head necrosis was well constructed, and the hydrogel was accurately implanted without any damage to the muscles, nerves, or blood vessels. All animals were healthy and no obvious signs of infection were observed. Several studies have reported the presence of osteogenic growth factors in PL [10, 20], and encapsulation of sPL in a hydrogel scaffolding enhances the retention capacity of constituent growth factors. As shown in Figure 2, the cross-sectional and longitudinal CT images show that PLS1 was partially repaired in the necrotic region, 4 weeks after the implantation, compared with the PLS0 group, whereas PLS2 showed a stronger therapeutic effect. Necrotic areas were still visible even after 12 weeks in the PLS0 groups but had been mostly reconstituted in rats treated with PLS1 and PLS2.

**Figure 3:**
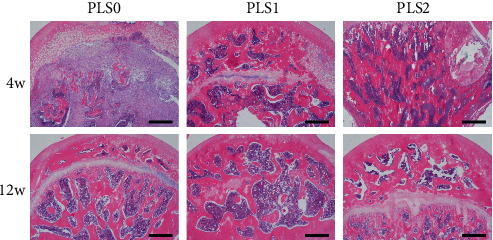
HE staining after treatment for femoral head necrosis (40x).

**Figure 4:**
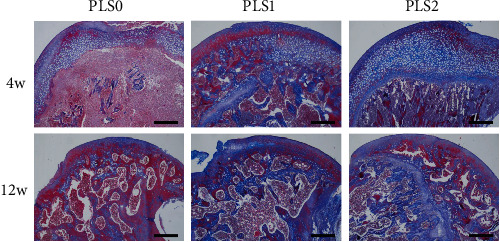
Histopathology Masson staining conducted 4 weeks and 12 weeks after ONFH treatment (40x).

**Figure 5:**
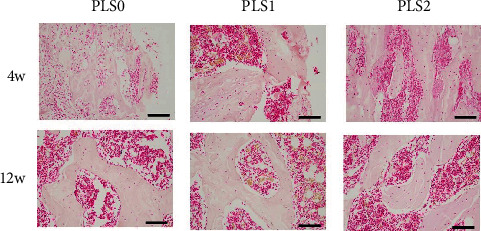
ALP staining of the histopathological osteogenic marker (200x).

**Figure 6:**
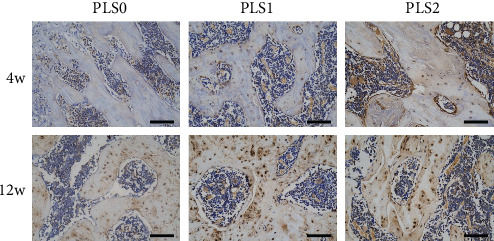
Histopathological type I staining demonstrated the composition of bone tissue (200x).

Histological examination of the femoral head necrosis showed that ONFH induction resulted in sparse cavities, diffuse spot-like bone marrow necrosis, and a sparse trabecular bone marrow cavity with large fat cells. Implantation of the hydrogels did not trigger any localized inflammatory reaction or fibrosis. PLS2 resulted in the formation of new bone and trabeculae by the 4^th^ week after implantation with causing osteonecrosis and hematopoietic necrosis. Likewise, PLS0 and PLS1 administration caused the formation of new bones but did not induce a trabecular arrangement (Figures 3 and 4). Consistent with the observations mentioned above, bone tissues expressing the osteogenic factor ALP and collagen I were observed in all groups, and the degree of positive staining increased in the PLS-implanted groups in a sPL concentration-dependent manner (Figures 5 and 6).

### 3.3. The sPL-Loaded Hydrogel Promoted Angiogenesis

**Figure 7:**
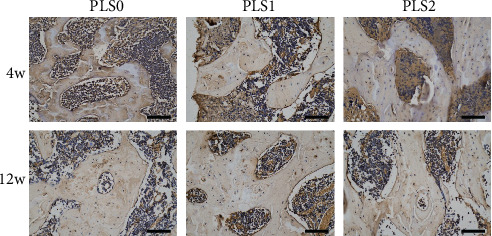
Histopathological CD31 staining demonstrating the formation of blood vessels in the femoral head (200x).

In addition to, the growth factors in sPL promote the adherence and proliferation of osteoblasts, while also facilitating angiogenesis via the proliferation of vascular endothelial cells. Platelet endothelial cell adhesion molecule-1 (CD31), which is typical for the endothelial lineage [21]. As shown in Figure 7, compared with the PLS0 group, CD31 staining increased in the PLS1 and PLS2 treatment groups during the 4^th^ week of treatment. The number of CD31-positive cells increased in a time and sPL concentration-dependent manner and peaked in the PLS2 treatment group, 12 weeks after surgery. Vadasz et al. [22] found that the decrease in VEGF (vascular endothelial growth factor, promote angiogenesis) levels was correlated with ONFH progression and promoted VEGF synthesis induced angiogenesis in the trabecular space in femoral head necrosis. Bai et al. [23] found that compared to BMP-2 alone, the combination of VEGF and BMP-2 increased the formation of the vascular networks and significantly enhanced the formation of ectopic bone. Consistent with previous reports, angiogenic factor CD31 was highly expressed in the PLS2-treated group (Figure 7).

### 3.4. The sPL-Loaded Hydrogel Inhibited Apoptosis in the Femoral Head Tissue

**Figure 8:**
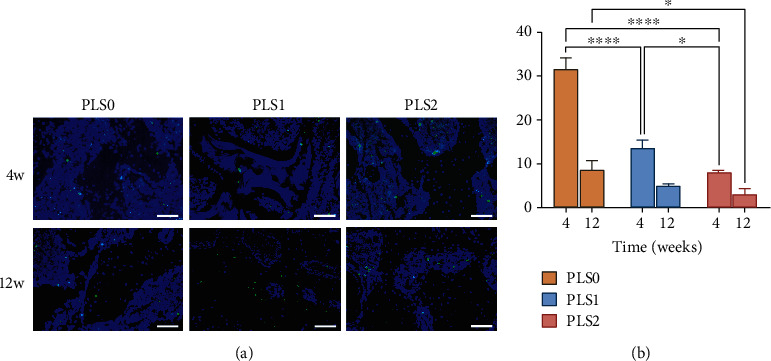
(a) Tunel staining conducted on the 4th week and 12th week after ONFH treatment (200x). (b) The apoptotic rate was calculated using the Tunel-staining results.

Numerous Tunel-positive apoptotic bone cells were observed in the necrotic femoral head, while the number of positive cells decreased significantly after hydrogel implantation. As shown in Figure 8, the rate of apoptosis in the PLS0, PLS1, and PLS2 groups were 31.48 ± 1.80%, 13.60 ± 1.27%, and 8.05 ± 0.27%, respectively, after 4 weeks, and 8.59 ± 1.55%, 4.97 ± 0.29%, and 3.08 ± 0.92%, respectively, after 12 weeks (Figure 8(b)). In the 4th week, compared with the PLS0 group, the apoptosis rate of the PLS1 and PLS2 groups was significantly reduced (*P* < 0.05). With the addition of sPL, the apoptosis rate in the tissues was also significantly reduced (*P* < 0.05). In the 12th week, compared with the PLS0 group, the apoptosis rate of PLS1 and PLS2 groups was significantly reduced (*P* < 0.05). With the addition of sPL, the apoptosis rate between PLS0 and PLS1 groups, and PLS1 and PLS2 groups had no significant statistical significance. In the PLS0 group, PLS1 group, and PLS2 group, compared with 4 weeks, the apoptosis rate in tissues at 12 weeks after operation was significantly reduced (*P* < 0.05).

ER stress-induced apoptosis can be inhibited by PRP exosomes in a manner independent of the PERK/CHOP pathway [24]. Bone mesenchymal stem cells (BMSCs) treated with PRP exosomes and dexamethasone showed enhanced phosphorylated Akt (protein kinase B) and Bad (Bcl-2 related death promoter) levels, as well as enhanced Bcl-2 expression, indicating that PRP exosomes can inhibit apoptosis by activating the Akt/Bad/Bcl-2 signaling pathway [24].

Despite the encouraging results, our study has two main limitations. First, we established an ONFH rat model in rats, which did not develop femoral head collapse. However, when we performed core decompression, the cartilage surface was partially destroyed, simulating the destruction of cartilage as a result of the femoral head collapse. The pathological condition of ONFH was not consistent with the actual clinical situation and also precluded the long-term effects of the use of the hydrogel. Therefore, at this stage, it is impossible to determine whether the hydrogel can prevent collapse. Instead, femoral collapse would have to be established in a large animal model to demonstrate cartilage repair. Second, we evaluated angiogenesis based only on CD31 expression levels, and microangiography can be used to observe the formation of new blood vessels in a more accurate manner.

## 4. Conclusion

Compared to thermosensitive hydrogel, the sPL-loaded thermosensitive hydrogel steadily released biological factors that reduced the level of osteoblast apoptosis, promoted osteogenesis and angiogenesis, and effectively prevented the development of ONFH in a rat model. Therefore, treatment using sPL-loaded thermosensitive hydrogel can be used in combination with core decompression surgery to improve the outcomes of femoral head necrosis treatment.

## Data Availability

The data used to support the findings of this study are included within the article.
